# Subclinical Arrhythmias and Conduction Disturbances in Children with Myopericarditis: A 24-hour Holter Monitorization Study

**DOI:** 10.5152/eurasianjmed.2022.22102

**Published:** 2022-10-01

**Authors:** Fuat Laloğlu, Naci Ceviz

**Affiliations:** 1 Division of Pediatric Cardiology, Department of Pediatrics, Atatürk University Faculty of Medicine, Erzurum, Turkey

**Keywords:** Children, myopericarditis, 24-hour Holter monitorization

## Abstract

**Objective:**

Myopericarditis is reported as a benign clinical entity in children and adolescents. However, there is no study investigating specifically the arrhythmias in these children. In the present study, we aimed to investigate the frequency of arrhythmias and conduction disturbances from surface electrocardiography and 24-hour Holter monitor, in children with myopericarditis.

**Materials and Methods:**

The medical records of the children with the diagnosis of myopericarditis between 2016 and 2021 were retrospectively reviewed. Clinical features, surface electrocardiography, and 24-hour Holter recordings were evaluated in terms of rhythm and conduction abnormalities.

**Results:**

Mean troponin level was 7980.52 ± 14880.27 ng/L at admission. At discharge, the mean troponin level was 8.27 ± 10.73 ng/L and it was above the upper limit of normal in 19/27 (70.4%) patients. Surface electrocardiography was normal in terms of arrhythmias and conduction disturbances in all patients. Twenty-four-hour Holter monitarization was found to be normal in 23 patients. In 4 patients, there were clinically insignificant arrhythmias; nonsustained accelerated idioventricular rhythm = 1, ventricular couplet with low rate = 1, infrequent supraventricular premature contractions = 1, and infrequent ventricular premature contractions + Wenckebach type atrioventricular block = 1.

**Conclusion:**

Our findings support that myopericarditis is a benign clinical problem in children also in terms of arrhythmia.

**Main Points**

- Myopericarditis is a clinically benign problem in children.
- Significant rhythm problems are not frequent in children with myopericarditis.
- Despite very high troponin levels, myocardial dysfunction is not seen.

## Introduction

Myopericarditis is primarily a pericarditic syndrome with some degree of myocardial involvement and a good clinical outcome.^1^ In adult patients with myopericarditis, frequency of arrhythmia has been reported as 65%, and most of them are atrial and ventricular premature contractions. The prevalence of significant arrhythmias is very low.^2^

There are a few number of studies investigating the clinical features of children with myopericarditis.^3-5^ In these studies, no significant arrhythmia has been reported. But, to our knowledge, there is no study investigating specifically the arrhythmias in children with myopericarditis. 

In present study, we aimed to investigate the frequency of arrhythmias and conduction disturbances from surface electrocardiography (ECG) and 24-hour Holter monitor in children with myopericarditis. 

## Materials and Methods

The study design was an observational single-center retrospective cohort study of patients admitted to the Pediatric Emergency Clinic of a university hospital. The medical records of the children with the diagnosis of myopericarditis between 2016 and 2021 are retrospectively reviewed. Clinical features (age, gender, weight, symptoms, and signs), surface ECGs, and 24-hour Holter recordings were evaluated in terms of rhythm and conduction abnormalities. Echocardiograms were reviewed, and the results of coronary imaging studies when available were recorded.

Myopericarditis was defined as a condition presenting with chest pain or pericardial friction rub, electrocardiographic findings of pericarditis (ST-T changes), elevated cardiac enzymes (troponin I or T and CK-MB), and normal left ventricular global systolic function.^3^ Patients with hemodynamically significant congenital heart disease, acute chest trauma, myocarditis, and only pericarditis with no myocardial involvement were excluded from the cohort analysis.

The study commenced following receipt of approval from the ethical committee of Atatürk University Faculty of Medicine (date: November 4, 2021, number of meetings: 7, decision number: 34), and parental consent or patient assent was not required.

At our center, all children presenting with chest pain suspected to be of cardiac etiology undergo electrocardiogram testing and they undergo cardiac enzymes measurement. If it is elevated, an echocardiography is being applied to evaluate the structural and functional cardiac abnormalities. Also, cardiac computerized tomography is obtained for differentiating the coronary abnormalities. In 25 patients, troponin I levels were measured, but in last 2 cases, center laboratory of the hospital changed the kits and began to measure troponin T levels. So, mean troponin levels were calculated from first 25 patients, and number of patients with elevated troponin level was given from whole group (27 patients).

## Results

A total of 27 children were included in the study. The mean age of the patients was 14.1 ± 2.7 years, and 20 were male (74%). All patients presented with chest pain (pressure-like = 6, stabbing = 9, burning = 1, unidentified = 11). Twelve patients defined additional complaints; palpitation = 2, back pain = 2, dyspnea = 2, numbness in left arm = 2, and others = 8. Ten patients (37%) had a history of upper respiratory tract infection (URTI) during the last 1 month of admission. 

In 15 children, ECG was normal on admission. In 7 patients, negative T wave and in 5 patients, pathologic ST elevation was detected. PR interval and corrected QT values were within normal limits in all children.

Echocardiography revealed normal left ventricular systolic functions in all patients. Segmental wall motion abnormality and pericardial effusion were not detected. In 2 patients, hemodynamically insignificant congenital heart diseases were diagnosed; small ventricular septal defect = 1, patent ductus arteriosus = 1.

In 25 patients, coronary computerized tomography was obtained. No coronary abnormality was detected. In one of them, myocardial bridging was suspected, but coronary angiography was normal. 

In 2 patients, symptoms were related to exercise. A treadmill test was performed and was evaluated as normal.

In only 7 patients, cardiac magnetic resonance imaging was possible and all were reported as compatible with myopericarditis. 

The mean troponin I levels are given in Table 1. In 14 patients, the troponin level at admission was at the peak value. In the remaining 13, troponin level reached the peak value at mean 22.52 ± 1736 hours of admission (range 2.7-51.52 hours). The mean hospitalization time was 84.66 ± 31.9 hours (range 21.68-142.42 hours). At discharge, the troponin level was above the upper limit of normal in 19/27 (70.4%) patients.

The mean erythrocyte sedimentation rate was 18 ± 12 mm/h, and C-reactive protein level was 19 ± 25 mg/dL. 

Twenty-four-hour ECG monitarization was found to be normal in 23 patients. In 4 patients, there were clinically insignificant arrhythmias; nonsustained accelerated idioventricular rhythm = 1, ventricular couplet with low rate = 1, infrequent supraventricular premature contractions = 1, infrequent ventricular premature contractions + Wenckebach type atrioventricular block = 1.

No significant deterioration in left ventricular functions was detected during the mean follow-up period of 1.89 ± 2.95 months. One patient presented with a similar clinical picture 1 year after the initial diagnosis and was diagnosed with recurrent myopericarditis.

## Discussion

Acute pericarditis indicates a pericardial inflammation with acute onset of symptoms, which usually last 4-6 weeks with treatment.^6^ Acute myocarditis indicates mainly a myocardial involvement with different degrees of depressed left ventricular systolic functions.^7^ Clinically, acute pericarditis and myocarditis commonly coexist.^1^ Myopericarditis, a new entity described in children in 2012,^3^ is defined as a primarily pericarditic syndrome with concomitant myocardial involvement and inflammation, and perimyocarditis specifies a primarily myocarditic syndrome with pericardial involvement.^1,6^

Clinically, myopericarditis is mostly defined as a condition presenting with chest pain or pericardial friction rub, ECG findings of pericarditis (ST-T changes), elevated cardiac enzymes such as troponin I and CK-MB, and normal left ventricular global systolic function.^3^

As in our study, it has been reported that myopericarditis mostly affects the adolescents.^3,5^ In the literature, there is a prominent male predominance.^5,8^ Also in our study, 74% of the patients was male. 

The reported main complaint in children with myopericarditis is chest pain.^3,5,8^ Non-specific findings such as decreased exercise capacity, palpitations, fever, or weakness may also be present.^5^ Among our patients also, the most frequent symptom (100%) was chest pain and 12 had additional symptoms. The frequency of a history of URTI was low (37%). 

Elevation in the troponin level is one of the main findings of patients with myopericarditis. Even in the patients with very high troponin levels, the prognosis of the disease is reported to be very good and does not seem to carry an adverse prognosis.^3,5^ Despite that, Babbitt et al^8^ reported a patient who was initially believed to have myopericarditis on admission but developed deterioration and required intubation. Repeat echocardiogram 22 hours after admission found that left ventricular ejection fraction (LVEF) had decreased to 37%. The final diagnosis reported as true myocarditis in this patient. This patient alerts us about that the patients with myopericarditis and true myocarditis may share the similar symptoms on initial admission. Despite the very high troponin levels (Table 1) on admission, during the hospitalization period, and short-term follow-up after discharge, none of our patients showed deterioration in LVEF. This finding supports the benign nature of the disease. 

In patients with myocardial injury due to any reason, arrhythmia may complicate the clinical status. In adult patients with myopericarditis, frequency of arrhythmia has been reported as 65%, and most of them are atrial and ventricular premature contractions. Prevalence of significant arrhythmias is very low.^2^

Although the clinical features of children with the diagnosis of myopericarditis had been reported in several studies,^3-5,8^ the frequency of arrhythmias had not been investigated in detail among children. To our knowledge, this is the first report investigating the frequency of arrhythmias in children with myopericarditis.

In children with true myocarditis (patients with recent signs and symptoms of heart failure with systolic dysfunction^9^), arrhythmias occur in up to 45% and include ventricular and atrial arrhythmias and high-grade atrioventricular block.^7^ Despite that, in children with myopericarditis, reported arrhythmia frequencies differ. Yoldaş and Örün^4^ did not report an arrhythmia or any type of conduction abnormality among 164 and Kobayasi^3^ in 12 children with myopericarditis. Depending on ECG findings, Başar et al^5^ reported ventricular premature beats in 5.1%, and supraventricular tachycardia in 2.6% of 39 children. Babbitt et al^8^ did not make a comment about the frequency of arrhythmias between children with myopericarditis and true myocarditis. In none of the above studies, 24-hour Holter monitorization had been performed. In our study, instead of the surface ECG, all patients were evaluated with 24-hour Holter monitorization, and in 4 patients, there were clinically insignificant arrhythmias; nonsustained accelerated idioventricular rhythm = 1, ventricular couplet with low rate = 1, infrequent supraventricular premature contractions = 1, infrequent ventricular premature contractions + Wenckebach type atrioventricular block = 1. This is an important new defined finding supporting that myopericarditis is a benign clinical problem in children also in terms of arrhythmia. 

In conclusion, our findings support that myopericarditis is a benign clinical problem in children. Also, in these patients, arrhythmia frequency is low and they are mostly insignificant.

### Study Limitations

The main limitations of the study are the relatively low number of patients and its retrospective nature. The findings need to be supported by evaluation of large number of patients.

## Figures and Tables

**Table 1.** Mean Troponin I Levels at Admission, at Peak Value, and at Discharge

	Mean Value (n = 25) ng/L Mean ± SD	Troponin Level/Upper Limit of Normal (n = 27) Mean ± SD (Minimum-Maximum)
At admission	7980.52 ± 14880.27	299.12 ± 405.41 (3.5-1873.75)
At peak value	9767.17 ± 15977.84	395.69 ± 395.69 (4.45-2000.00)
At discharge	211.13 ± 341.16	8.27 ± 10.73 (0.06-39.25)

SD, standard deviation.
